# Investigation of the influence of Arg555Trp and Thr538Pro TGFBI mutations on C-terminal cleavage and cell endoplasmic reticulum stress

**Published:** 2012-05-03

**Authors:** Miaomiao Zhu, Ping Yu, Bo Jiang, Yangshun Gu

**Affiliations:** 1 Department of Ophthalmology, Ophthalmic Genetic Research Centre, The First Affiliated Hospital, School of Medicine, Zhejiang University, Hangzhou, China; 2 Department of Medical Genetics, School of Medicine, Zhejiang University, Hangzhou, China

## Abstract

**Purpose:**

To gain insight into the mechanisms underlying the transforming growth factor-beta induced (TGFBI)-related corneal dystrophies and the influence of the Arg555Trp and Thr538Pro, TGFBI mutations on C-terminal cleavage and cell endoplasmic reticulum (ER) stress were investigated.

**Methods:**

The Arg555Trp and Thr538Pro mutations known to be associated with corneal dystrophy granular type I and lattice corneal dystrophy, respectively, were introduced with the two-sequential PCR site-directed mutagenesis technique. Wild-type and mutant *TGFBI* DNAs were cloned into the pcDNA3.1(-)/myc-his expression vector and overexpressed in HeLa and human corneal epithelial cells (HCE) with transient transfection. Transfection efficiency was measured by the expression of green fluorescent protein. Expression of the fusion proteins was measured with western blot analysis with anti-c-myc-tag and anti-TGFBI antibodies. For cell ER stress studies, the expression levels of GRP78/BiP in HeLa cells were analyzed with western blot analysis using an anti-GRP78 monoclonal antibody at 12, 24, and 48 h after either the wild-type or mutant plasmid was transfected.

**Results:**

Arg555Trp and Thr538Pro mutant TGFBIp were detected with the anti-c-myc and anti-TGFBI antibodies, while wild-type TGFBIp was detected only with the anti-TGFBI antibody, indicating that the Arg555Trp and Thr538Pro mutations prevent the C-terminal cleavage of TGFBIp. Moreover, no significant differences were seen in the expression levels of GRP78/BiP between the mutant and wild-type TGFBIp groups, suggesting that mutations in TGFBIp are unlikely to disrupt protein folding or induce cell ER stress.

**Conclusions:**

This is the first time that the influence of TGFBI mutants on C-terminal cleavage and cell ER stress has been illustrated. Corneal dystrophy–related mutations are more likely to disrupt the interaction of TGFBI with critical binding proteins than affect the whole protein structure.

## Introduction

Corneal dystrophies (CDs) are a heterogeneous group of diseases characterized by bilateral, non-inflammatory deposits in corneal layers, which result in corneal opacification and visual acuity impairment. Transforming growth factor-beta induced (TGFBI)-related corneal dystrophies arise from heterozygous mutations in the transforming growth factor-beta induced gene (*TGFBI*), which is located on human chromosome 5q31. According to the new international classification of corneal dystrophies, it belongs to category 1 (clinically and histologically well defined dystrophy with identification of the gene and the mutations) and includes two phenotypically distinct CDs: lattice corneal dystrophy (LCD) and granular corneal dystrophy (GCD) [1,2]. More than 30 distinct *TGFBI* mutations have been identified in CDs [3], among which c.1663C>T (p.Arg555Trp) is the most frequent mutation resulting in GCD I according to several studies among various ethnic groups [4-9]. Recently, a novel c.1613A>C (p.Thr538Pro) mutation associated with LCD was identified by our group [10].

**Figure 1:**
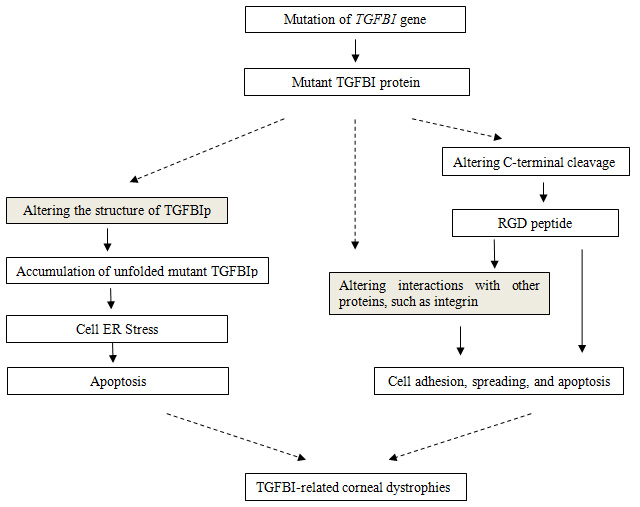
Schematic of proposed mechanism of TGFBI-related corneal dystrophies in present study. The hypothesis of pathogenesis of TGFBI-related corneal dystrophies mainly consists of two aspects: altering the binding interactions of TGFBIp with other critical proteins and interfering with the TGFBIp folding. The influence of *TGFBI* mutations on C-terminal cleavage and cell endoplasmic reticulum stress investigated in the present study was based on the aspects of the hypothesis. The mechanism by which these mutations cause disease remains unknown, as indicated by the dashed arrow.

The TGFBI protein (TGFBIp, also known as keratoepithelin or βig-H3), which is encoded by the *TGFBI* gene, is composed of 683 amino acids and has a molecular mass of approximately 68 kDa. The protein is an extracellular matrix (ECM) protein that contains an N-terminal secretory signal peptide (1–23 aa), a cysteine-rich EMI domain (45–99 aa), four repeats of fasciclin-1-like (FAS1) domain (136–634 aa), and a C-terminal arginine-glycine-aspartic acid (RGD) sequence (642–644 aa). Most of the mutations that have been identified are located in the fourth FAS1 domain [3], such as p.Arg555Trp and p.Thr538Pro. Although the mechanism by which these mutations cause disease remains unknown, the hypothesis of pathogenesis mainly consists of two aspects: altering the binding interactions of TGFBIp with other critical proteins and interfering with TGFBIp folding (Figure 1).

RGD, an integrin ligand, plays an important role in cell adhesion, spreading, and apoptosis [11]. Wild-type TGFBIp undergoes C-terminal cleavage beside the RGD sequence after secretion, which is likely to leave the RGD exposed for interactions [11-13]. Cleavage is thought to be an important step in TGFBI processing and allows TGFBIp to perform its biologic function. However, to the best of our knowledge, there is no direct evidence regarding this proteolytic processing in mutant TGFBI proteins.

Structure modeling studies predict that certain mutations of *TGFBI* disrupt the TGFBIp structure, thereby leading to misfolding of the protein [7,14]. Accumulation of unfolded proteins causes endoplasmic reticulum (ER) stress and triggers an ER self-defense response called the unfolded protein response (UPR), which ultimately decreases the amount of unfolded proteins [15]. If the protective mechanisms are not efficient enough to decrease the amount of misfolded proteins and restore normal ER function, the cells will die via apoptosis. Glucose-regulated protein (GRP78, also known as BiP) is the major chaperone for ER stress and plays a key role in protein folding [16,17]. Evidence is emerging that ER stress–related toxicity plays a causative role in disease development; this is especially true for diseases caused by missense mutations in certain genes [18-22]. It is therefore necessary to investigate the effect of mutant TGFBIp on cell ER stress, thereby shedding light on the pathogenesis of TGFBI-related corneal dystrophies.

Based on the two hypotheses, we investigated C-terminal cleavage and cell ER stress in cells expressing with two mutant TGFBI proteins, c.1663C>T (p.Arg555Trp) and c.1613A>C (p.Thr538Pro), which would result in GCD I and LCD, respectively. The results show that the Arg555Trp and Thr538Pro mutant TGFBI proteins are resistant to C-terminal cleavage. Furthermore, the overexpressed Arg555Trp and Thr538Pro mutant TGFBI proteins were unlikely to induce cell ER stress. This is the first time the influence of mutant TGFBI proteins on C-terminal cleavage and cell ER stress has been illustrated. These findings shed light on and increase understanding of the pathogenic mechanisms of TGFBI-related corneal dystrophies.

## Methods

### Plasmid construction

The *TGFBI* cDNA clone was generated with reverse transcription–polymerase chain reaction (RT–PCR) from RNA isolated from a human HeLa cell line using the primers TGFBI-WT-F and TGFBI-WT-R (Table 1) and the SuperScript First-Strand Synthesis System for RT–PCR kit (Invitrogen, Carlsbad, CA) following the manufacturer’s instructions. Using these primers, the *TGFBI* stop codon was removed, and 5′-terminal Xho I/3′-terminal Hind III restriction enzyme recognition sites were added. After double digestion with Xho I/Hind III, the DNA products were ligated into the pcDNA3.1(-)/myc-his vector (Invitrogen) to construct the pcTGFBI-WT-myc plasmid.

**Table 1:** Primers used for PCR.

**Amplified fragments**	**Primer name**	**Primer sequence (5′>3′)**
TGFBI-WT	TGFBI-WT-F	GTCACTCGAGTCGGTCGCTAGCTCGCTC
	TGFBI-WT-R	GTCAAAGCTTCTATGCTTCATCCTCTCTAATAAC
TGFBI-T538P-I	TGFBI-WT-F	GTCACTCGAGTCGGTCGCTAGCTCGCTC
	TGFBI-538-R	CAAAGACTGG*GTAGACTCCT
TGFBI-R555W-I	TGFBI-WT-F	GTCACTCGAGTCGGTCGCTAGCTCGCTC
	TGFBI-555-R	AGTCTGCTCCA*TTCTCTTGG
TGFBI-T538P-II	TGFBI-538-F	AGGAGTCTACC*CAGTCTTTG
	TGFBI-WT-R	GTCAAAGCTTCTATGCTTCATCCTCTCTAATAAC
TGFBI-R555W-II	TGFBI-555-F	CCAAGAGAAT*GGAGCAGACT
	TGFBI-WT-R	GTCAAAGCTTCTATGCTTCATCCTCTCTAATAAC
TGFBI	TGFBI-qPCR-F	AGGACTGACGGAGACCCTCAAC
	TGFBI-qPCR-R	TCCGCTAACCAGGATTTCATCAC
GAPDH	GAPDH-F	CAGGGCTGCTTTTAACTCTGG
	GAPDH-R	TGGGTGGAATCATATTGGAACA

*: site of introduced mutation.

**Figure 2:**
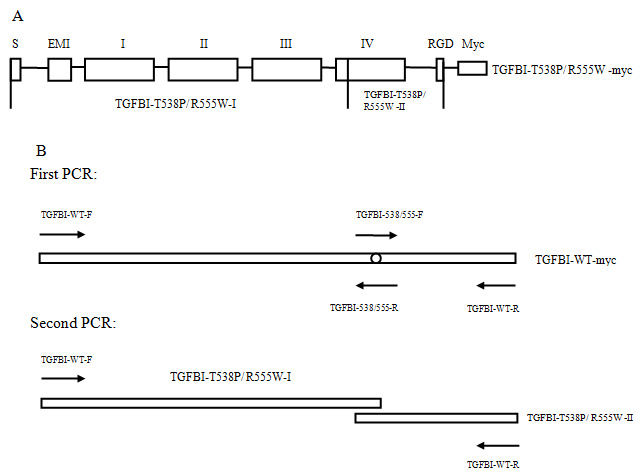
Schematic representation of the recombinant mutant TGFBI proteins and the principal of the two-sequential PCR site-directed mutagenesis technique. **A**: S: signal sequence, EMI: cysteine-rich EMI domain, I to IV: four FAS1 domains, RGD: Arg-Gly-Asp domain, Myc: myc tag protein, *TGFBI-T538P-I*: bp 1 to 1613 (the 538 mutant site), *TGFBI-R555W-I*: bp 1 to 1663 (the 555 mutant site), *TGFBI-T538P/R555W-II*: from the mutant site to the 3′ end of the sequence. **B**: The rectangle represents the template DNA, the circle in the rectangle represents the mutant site, and the arrow represents the primer. In the first PCR, *TGFBI-T538P/ R555W-I* were amplified with the TGFBI-WT-F and TGFBI-T538P/ R555W-R primers. *TGFBI-T538P/ R555W-II* were amplified with the TGFBI-T538P/ R555W-F and TGFBI-WT-R primers. In the second PCR, full-length *TGFBI-T538P/ R555W* were amplified with the TGFBI-WT-F and TGFBI-WT-R primers.

The mutations (T538P and R555W) were introduced with the two-sequential PCR site-directed mutagenesis technique (Figure 2). In the first PCR, the TGFBI-T538P/R555W-I and TGFBI-T538P/R555W-II fragments were amplified individually with pcTGFBI-WT-myc as a template. In the second PCR, full-length mutant TGFBI was amplified by primers TGFBI-WT-F and TGFBI-WT-R with purified TGFBI-T538P/R555W-I and II as the DNA templates. PCR was performed in a 50 μl reaction mixture containing 100 ng template DNA, 1 μM of each primer (Table 1), and 2.5 U DNA Polymerase (AccuPrime Taq DNA Polymerase High Fidelity, Invitrogen) in the 1× PCR buffer provided by the manufacturer. The thermocycling program was as follows: initial denaturation at 94 °C for 5 min; followed by 30 cycles of denaturation at 94 °C for 1 min, annealing at 65 °C for 1 min, and elongation at 68 °C for 3 min (for >1 kb PCR product) or 1.5 min (for <1 kb PCR product); and 5 min of final extension at 68 °C. The PCR products were gel-purified using the AxyPrep DNA Gel Extraction Kit (Axygen, Union City, CA) following the manufacturer’s instructions. After double digestion with Xho I/Hind III, the purified products were ligated into the pcDNA3.1(-)/myc-his vector to construct the pcTGFBI-T538P/R555W-myc plasmids. The plasmids were transformed into DH5а chemically competent cells and seeded onto 1% LB agar plates supplemented with ampicillin. After 18 h of incubation, 12 colonies were picked from each sample and cultured in LB medium with 200 rpm/min shaking overnight. The plasmid DNA was extracted using the Qiagen Plasmid Midi Kit (Qiagen, Hilden, Germany) following the manufacturer’s instructions. The sequences of all of the constructs were verified with direct sequencing.

### Cell culture and transient transfection

HeLa cells were cultured in DMEM medium containing 10% fetal bovine serum (FBS; Invitrogen) and antibiotics. Human corneal epithelial (HCE) cells were grown in DMEM/F12 medium containing 10% FBS, 5 µg/ml insulin (Sigma, St. Louis, MO), 10 ng/ml epidermal growth factor (EGF; Invitrogen), and antibiotics. All cells were incubated at 37 °C in a humidified atmosphere containing 5% CO_2_, and 0.8 or 2×10^6^ cells were seeded onto 60 mm or 100 mm dishes and grown for 18 h (HeLa) or 22 h (HCE) in antibiotic-free medium before transfection. When the cells were grown to 90%–95% confluence, the cultures were transfected with 4.5 µg *TGFBI* plasmids and 4.5 µg pEGFP-N1 vector (Clontech, Mountain View, CA) using 30 µl Lipofectamine 2000 (Invitrogen) in 12 ml serum-free medium (100 mm dish) according to the manufacturer’s instructions. The cells were incubated at 37 °C in a 5% CO_2_ incubator for 4 h, and then the medium was replaced with fresh culture medium containing 10% FBS. The transfection efficiency was measured by the expression levels of the enhanced green fluorescent protein, which was visualized directly with fluorescence microscopy.

For cell ER stress study, tunicamycin (T7765; Sigma) was chosen as a positive inducer [23]. The expression levels of GRP78 proteins were examined 18 h after treatment with or without 5 μg/ml tunicamycin. Dimethyl sulphoxide (DMSO) was used as solvent.

### Western blotting

For extracellular protein analysis, the culture medium was replaced with serum-free medium 48 h post-transfection to eliminate the interference of TGFBIp from FBS. Another 48 h later, the serum-free medium was collected and concentrated with 15 centrifugal filter devices (Amicon Ultra 30K NMWL device; Millipore, Boston, MA) according to the manufacturer’s instructions. For the intracellular protein analysis, the cells were harvested at 12, 24, and 48 h after transfection. After being washed with 1× PBS, cells were lysed with a complete lysis kit (Roche, Basel, Switzerland) following the instruction manual. Protein concentrations were determined using a BCA Protein Assay Kit (Thermo Scientific, Waltham, MA). For sodium dodecyl sulfate PAGE (SDS–PAGE), 5× SDS was diluted 1:4 with the protein extract, and the samples were boiled for 10 min. Equal amounts of protein were subjected to electrophoresis on 8% SDS–PAGE gels and transferred to a polyvinylidene difluoride membrane (Millipore, Boston, MA). The membrane was blocked with 5% nonfat dry milk in 1× Tris-buffered saline buffer containing 0.1% Tween-20 (TBST) for 2 h at room temperature and then incubated overnight at 4 °C with primary antibody. After being washed with 1× TBST for 30 min, the membrane was incubated with the secondary antibody for 1 h at room temperature.

A mouse anti-c-myc-tag monoclonal antibody (1:750; GenScript, Piscataway, NJ) and a rabbit anti-TGFBIp polyclonal antibody (1:750; Santa Cruz Biotechnology, Santa Cruz, CA) were used to detect the TGFBI-myc fusion protein. Mouse anti-GRP78 monoclonal antibody (1:500; Santa Cruz Biotechnology) was used to detect GRP78. Goat antimouse immunoglobulin (IgG; H&L; [HPR] 1:2,000; GenScript) and goat antirabbit IgG (HPR; 1:2,000; Santa Cruz Biotechnology) were used as secondary antibodies. Rabbit anti-β-actin antibody (1:2,000; Santa Cruz Biotechnology) was used to determine the amount of β-actin to ensure equal amounts of protein were loaded in each lane for electrophoresis. Immunostaining was visualized with enhanced chemiluminescence (ECL, Biologic Industries, Beit Haemek, Israel). The signal intensity of the bands was analyzed with Quantity One 1-D analysis software (Bio-Rad, Hercules, CA).

### Quantitative real-time reverse transcription polymerase chain reaction

Total RNA was extracted from HCE and HeLa cells using RNAiso Plus (TaKaRa, Dalian, China) according to the manufacturer’s protocol. cDNA was synthesized by reverse transcription using the PrimeScript RT Reagent Kit (TaKaRa). RT–PCR was performed in a total volume of 20 μl reaction mixture containing 500 ng total RNA, 4 μl 5× PrimeScript Buffer, 1 μl PrimeScript RT Enzyme Mix I, 1 μl oligo dT primer (50 μM), and 1 ml Random 6 mers (100 μM). The mixture was incubated at 37 °C for 15 min followed by 5-s inactivation at 85 °C. Real-time PCR was performed in a 10 μl reaction mixture containing 5 μl SYBR Premix Ex Taq, 0.2 μl of each primer (10 μM; Table 1), 0.2 μl ROX reference dye, and 1 μl of the cDNA sample. Amplification was performed on an ABI 7900 instrument (Applied Biosystems, Foster City, CA). The thermocycling program included initial denaturation at 95 °C for 10 min, followed by 40 cycles of 95 °C for 5 s and 60 °C for 30 s. Glyceraldehydes 3-phosphate dehydrogenase (*GAPDH*) was selected as a reference gene. Data were analyzed using the comparative cycle threshold method.

### Statistical analyses

The post-hoc test for multiple comparisons after one-way ANOVA was Tukey test was used to determine the intensity ratio differences. A p<0.05 was used to determine significant differences between the groups.

## Results

### Mutant plasmids construction

**Figure 3:**
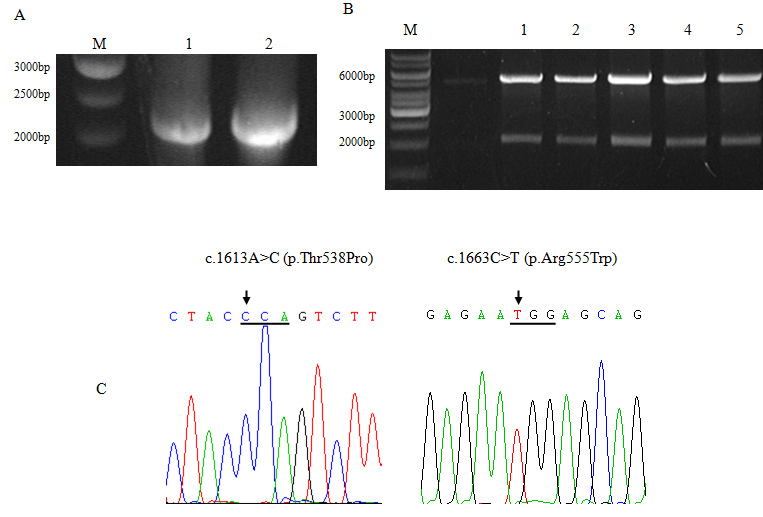
Construction of mutant plasmids. **A**: PCR products of the recombinant mutant TGFBI-T538P and TGFBI-R555W genes. M: 1 kb marker (Fermentas, Burlington, Canada), lane 1: TGFBI-T538P, lane 2: TGFBI-R555W. **B**: Constructed pcTGFBI-WT/T538P/R555W-myc plasmids were double digested into two bands approximately 2 kb and 5.5 kb, respectively, using Xho I and Hind III. M: 1 kb marker, lane 1: pcTGFBI-WT-myc, lane 2–lane 3: pcTGFBI-T538P-myc, lane 4–lane 5: pcTGFBI-R555W-myc. **C**: Right: Partial sequence of the pcTGFBI-T538P plasmid showing the A to C change at position c.1613 (the forward strand is shown) resulting in the p.Thr538Pro mutation (underlined). Left: Partial sequence of the pcTGFBI-R555W plasmid showing the C to T mutation at position c.1663 (the forward strand is shown) resulting in the p.Arg555Trp mutation (underlined). The arrow points toward the position of the mutations.

To construct the mutant pcTGFBI-T538P/R555W-myc plasmid, the two-sequential PCR site-directed mutagenesis technique was used. Agarose gel electrophoresis showed a clear band with a size corresponding to approximately 2 kb after the second PCR (Figure 3A; lanes 1, 2). This corresponded with the size of the full-length *TGFBI* cDNA. The wild-type and mutant pcTGFBI plasmids were double digested with Xho I/Hind III. As expected, each plasmid was cut into 2-kb and 5.5-kb fragments (Figure 3B). The sequences of all the constructs were also verified with direct sequencing. The results clearly showed that A was replaced with C at position c.1613 of the T538P plasmid, while T substituted C at c.1663 of the R555W plasmid (Figure 3C). Aside from the induced point mutation, there were no other mutations throughout the entire sequence.

### Arg555Trp and Thr538Pro mutant TGFBI proteins resist C-terminal cleavage

**Figure 4:**
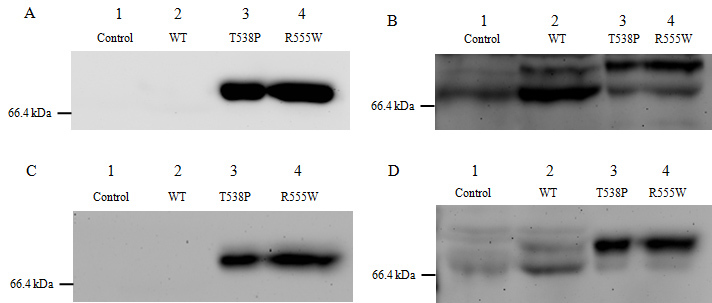
Western blot analysis of the recombinant wild-type and mutant proteins. HCE and HeLa cells (**A**, **B**: HCE cell; **C**, **D**: HeLa cells) were transfected with wild-type (lane 2) and two mutant TGFBI plasmids (lanes 3 and 4) or were not transfected plasmid (lane 1). The culture medium was replaced with serum-free medium 48 h post-transfection. Another 48 h later, the serum-free media from overexpressed cells were collected and concentrated. **A** and **C:** 65 µg protein was loaded into each well. The TGFBI-T538P-myc and TGFBI-R555W-myc protein were detected with an anti-c-myc-tag antibody (lanes 3 and 4), while the wild-type TGFBI-myc protein was not (lane 2). **B** and **D**: 65 µg protein was loaded on to each well and detected with an anti-TGFBIp antibody. The wild-type and mutant TGFBI-myc fusion proteins were detected as two bands of different intensities (lanes 2–4). Endogenous TGFBIp is shown in the control lane (lane 1). The stronger bands in lanes 3 and 4 represent full-length mutant TGFBI-myc proteins, while the stronger band in lane 2 represents the wild-type TGFBI-myc protein that lacks its C-terminus. There was an approximate 10 kDa difference between the full-length and cleaved proteins.

To investigate the expression of the wild-type and mutant TGFBI-myc fusion proteins, HCE and HeLa cells were used for transient plasmid transfection. Each protein expressed a Myc tag at its C-terminus. In Figure 4A, 65 µg protein from the media of HCE cells was loaded into each well. As expected, the wild-type TGFBI-myc protein was not detected by an anti-c-myc-tag antibody due to C-terminal cleavage (lane 2). This was consistent with Kim et al.’s study, which indicated that wild-type TGFBI undergoes C-terminal cleavage processing after secretion [11]. Surprisingly, TGFBI-T538P-myc (lane 3) and TGFBI-R555W-myc (lane 4) fusion proteins were detected by anti-c-myc-tag antibody at approximately 70 kDa.

The same samples were immunoblotted with an anti-TGFBI antibody (Figure 4B). First, the wild-type and mutant type TGFBI-myc fusion proteins were detected as two bands of different intensities (lanes 2–4). An endogenous TGFBIp was seen in the control lane (lane 1), but it was much weaker than the recombinant wild-type TGFBIp. Second, the smaller wild-type TGFBIp band was much stronger than the larger one, while the opposite was observed for the mutant TGFBI protein. Furthermore, there was an approximately 10 kDa difference between the two bands, indicating that the cleavage site may be 10 kDa away from the C-terminal end of the protein.

These results were replicated in HeLa cells (Figure 4C,D), reinforcing that the mutant TGFBI escapes C-terminal cleavage after secretion.

### Effect of mutation and cell type on TGFBIp expression

**Figure 5:**
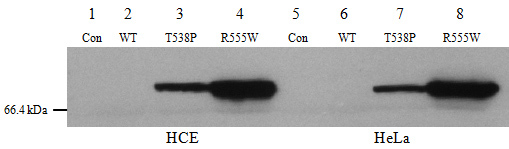
Comparison of the amount of recombinant TGFBI secreted from HCE and HeLa cells. HCE and HeLa cells (lane 1–lane 4: HCE, lane 5–lane 8: HeLa cells) were transfected with wild-type (WT) and the two mutant TGFBI plasmids. Control represents HCE or HeLa cells without transfection with plasmids. Media from overexpressed cells were collected and analyzed with an anti-myc antibody. The expression level of the R555W mutant protein was much higher than that of the T538P mutant protein. Moreover, the HCE cells expressed more full-length mutant protein than the HeLa cells. This result indicates the cell type and mutation affect the TGFBIp expression level.

To compare the expression level of TGFBIp-myc in HCE and HeLa cells, an equal amount of protein (70 μg) was collected from the media of these cells after transfection and subjected to electrophoresis. The blot showed that there was more full-length TGFBI-T538P/R555W-myc protein in HCE cells compared with HeLa cells (Figure 5). In addition, the endogenous levels of the *TGFBI* gene expressed in both cell lines were checked with quantitative real-time RT–PCR. The results showed the expression levels of the endogenous *TGFBI* gene of the HCE cells were higher than those of the HeLa cells (data not shown). Moreover, the expression levels of the TGFBI-R555W-myc protein were higher than those of the TGFBI-T538P-myc protein, indicating that the mutation also had an effect on protein expression.

### Arg555Trp and Thr538Pro mutant TGFBI proteins are unlikely to induce endoplasmic reticulum stress

**Figure 6:**
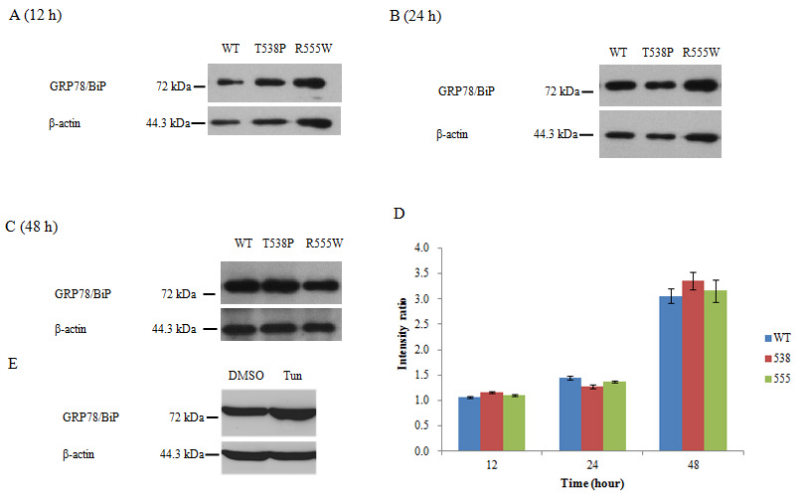
Western blot analysis of GRP78/BiP. HeLa cells were harvested at 12 (**A**), 24 (**B**), and 48 (**C**) h after transfection with the pcTGFBI-WT-myc (WT), pcTGFBI-T538P-myc (T538P), and pcTGFBI-R555W-myc (R555W) plasmids. The proteins were separated on 8% SDS-polyacrylamide gels, transferred, and probed with an anti-GRP 78 antibody. The amount of β-actin was analyzed with an anti-β-actin antibody to ensure that equal amounts of protein were loaded in each lane for electrophoresis. The intensity ratio was calculated by the intensity of the GRP78 band divided by the intensity of the β-actin band (**D**). No significant differences were found between the mutant and wild-type groups at 12 and 48 h (p>0.05). The intensity ratio of the T538P group was slightly lower than the wild-type group at 24 h (p=0.003). This transient fluctuation returned to the same level as the wild-type group after 48 h. The expression of GRP78/BiP at 48 h was significantly higher than that at 12 and 24 h (p<0.05). In the positive control group, GRP78/BiP expression was significantly increased in the tunicamycin-treated cells compared with the untreated cells (**E**).

The expression of the ER stress molecular chaperone GRP78/BiP was analyzed to investigate ER stress after transfection. Intracellular proteins were collected from HeLa cells at 12, 24, and 48 h after transfection with different plasmids. An equal amount of protein was subjected to electrophoresis. No significant differences were found between the mutant and wild-type groups at 12 and 48 h (p>0.05; Figure 6A, C,D). The intensity ratio of the T538P group was slightly lower than the wild-type group at 24 h (p=0.003; Figure 6B,D). However, this transient fluctuation returned to the same level as the wild-type group after 48 h. The expression of GRP78/BiP at 48 h was significantly higher than that at 12 and 24 h (p<0.05; Figure 6). In the positive control group, GRP78/BiP expression was significantly increased in the tunicamycin-treated cells compared with the untreated cells (Figure 6E).

## Discussion

To gain insight into the mechanism underlying TGFBI-related corneal dystrophies, we first investigated the influence of the Arg555Trp and Thr538Pro TGFBI mutant proteins on C-terminal cleavage and ER stress. In this study, we demonstrated that the Arg555Trp/Thr538Pro mutations prevent the C-terminal cleavage of TGFBIp.

The recombinant wild-type TGFBI-myc fusion protein was detected only by an anti-TGFBI antibody but not by an anti-myc antibody (Figure 4, lane 2). This demonstrated that the wild-type TGFBI underwent C-terminal cleavage after secretion, and the major form of TGFBIp in the medium lacked its C-terminal. The control lane without any plasmid transfection (Figure 4B, D, lane 1) also showed a faint band when detected by the anti-TGFBI antibody. This could be because HeLa or HCE cells secrete endogenous TGFBIp but at much lower levels than the recombinant wild-type TGFBIp. In addition, the size of the weak band was the same as that of recombinant wild-type TGFBIp, indicating that the endogenous TGFBIp also undergoes C-terminal processing. These results were consistent with previous studies. A study previously reported that recombinant wild-type TGFBIp expressed in Chinese hamster ovary cells lacked its RGD sequence, suggesting a C-terminal processing reaction [13], which was later demonstrated by a Korean research group [11]. An in vivo study also found that the majority of TGFBIp in normal human corneas was C-terminally truncated after the RGD sequence [12].

Moreover, we found an exciting phenomenon: the Arg555Trp and Thr538Pro mutant TGFBIp was detected by an anti-Myc antibody and an anti-TGFBI antibody (Figure 4, lanes 3, 4). When proteins were immunoblotted with the anti-TGFBI antibody, two bands with different intensities were seen in each lane (Figure 4B, D, lanes 3, 4). The stronger band represented the full-length approximately 70 kDa mutant TGFBIp. The weaker band represented endogenous TGFBIp because the intensity was the same as the control lane. This indicated that the Arg555Trp and Thr538Pro mutant TGFBIp was resistant to C-terminal cleavage, and the full-length mutant TGFBIp was the major form in the medium. This is the first time the difference between the C-terminal cleavage of mutant and wild-type TGFBIp has been shown.

The C-terminal cleavage of wild-type TGFBIp is thought to be a heterogeneous event leading to a truncated C-terminus [12]. However, the cleavage always occurs either upstream or downstream of the integrin-binding RGD peptides. In this study, the RGD peptide was approximately 7 kDa from the C-terminus of the recombinant fusion protein. There was a difference of approximately 10 kDa between the full length and the cleavage proteins (Figure 4B,D), which supports the idea that the cleavage occurred near the RGD peptide. If the cleavage occurred before the RGD, the C-terminal fragment released from TGFBI containing the RGD sequence would mediate apoptosis [11]. On the contrary, if the processing occurred after the RGD sequence, it may make the area around the RGD peptide more accessible, thereby triggering the integrin binding activity [12,24]. Integrins are functional receptors that mediate the attachment of cells to the ECM or cell-cell interaction by recognizing certain sequences, such as the RGD peptide. A study reported that TGFBIp mediates HCE cell adhesion through α_3_β_1_ integrin [25]. According to these studies, C-terminal processing is an important event in TGFBIp function. We suspected that the mutations preventing TGFBIp cleavage would disrupt the interaction between TGFBIp and integrin, thereby resulting in disease development (Figure 1).

Secondary structural predictions show that mutations possibly leading to misfolding of TGFBIp would result in complete structural disruption [14,26,27]. The ER is the first compartment in the secretory pathway. All secretory proteins enter the ER for synthesis, modification, and folding [15]. After translation, TGFBIp is transported into the ER guided by the N-terminal signal sequences. After folding in the ER, TGFBIp is transported to the Golgi for further modifications. This processing was demonstrated with immunofluorescence microscopy in a recent study [28]. Accumulation of misfolded proteins increases cell ER stress, which has been found to be associated with disease development. A study on diabetic retinopathy showed that a high concentration of glucose increases ER stress and induces the UPR in retinal pericytes [29]. Another study reported that accumulation of mutant Rh1 (rhodopsin-1) protein increases cell ER stress in a fly model for Rhodopsin^Pro23His^ human retinitis pigmentosa [21]. Overexpression of mutant superoxide dismutase 1 (SOD1), which could result in amyotrophic lateral sclerosis, causes the upregulation of GRP78/BiP expression in COS7 cells and transgenic mice [30]. Investigation of the influence of TGFBI mutations on cell ER stress would help to characterize the mechanisms underlying TGFBI-related corneal dystrophy. GRP78/BiP, the major ER stress molecular chaperone, is a peptide-dependent ATPase that binds to UPR transducers under non-stressed conditions. When misfolded proteins accumulate in the cells, GRP78/BiP dissociates from the UPR transducers to chaperone these misfolded proteins and activates one or more transducers simultaneously [22]. This is the first event in signal transduction in response to the accumulation of unfolded proteins in the ER [15].

In this study, we analyzed the GRP78/BiP expression levels of HeLa cells at 12, 24, and 48 h after transfection with the pcTGFBI-WT/T538P/R555W-myc plasmids. No significant differences were found between the mutant and wild-type groups at 12 and 48 h (p>0.05; Figure 6A,C,D). The intensity ratio of the T538P group was slightly lower than the wild-type group at 24 h (p=0.003; Figure 6B,D). However, this transient fluctuation returned to the same level as the wild-type group after 48 h. This result indicated that the p.Arg555Trp and p.Thr538Pro mutant TGFBI proteins do not upregulate or downregulate GRP78/BiP expression compared with the wild-type TGFBIp, suggesting that these two mutations of TGFBIp were unlikely to disrupt protein folding or that the disruption could be repaired by the UPR in the cells. This was consistent with the Kim et al.’s [31] study, which reported that the mutant forms of TGFBI commonly found in TGFBI-linked corneal dystrophies did not significantly affect the fibrillar structure visualized with electron microscopy. They suggested it was unlikely that a single point mutation of TGFBI significantly affects the whole protein structure.

In conclusion, we demonstrated that Arg555Trp and Thr538Pro mutations prevent TGFBIp from C-terminal cleavage and are unlikely to induce cell ER stress. This is the first time that a difference between the C-terminal cleavage of mutant and wild-type TGFBIp has been demonstrated. Although the mechanism underlying TGFBI-related corneal dystrophies is still unclear, we suspect that corneal dystrophy–related mutations are more likely to disrupt the interaction of TGFBI with critical binding proteins rather than altering the entire protein structure.
